# EMERGENCE OF NEW STRAINS OF SARS-COV-2: AFRICA’S FATE AND ITS PREPAREDNESS AGAINST COVID-19 INFECTION WAVES

**DOI:** 10.21010/Ajid.v16i2.1

**Published:** 2022-05-06

**Authors:** Ohia Chinenyenwa M.D, Bakarey Adeleye Solomon, Ahmad Tauseef, Haroon Haroon, Ana Godson R. E. E., Sridhar Mynepalli K.C.

**Affiliations:** † Department of Environmental Health Sciences, Faculty of Public Health, College of Medicine, University of Ibadan, Nigeria; †† Institute for Advanced Medical Research and Training (IAMRAT), College of Medicine, University of Ibadan, Nigeria; ††† Department of Epidemiology and Health Statistics, School of Public Health, Southeast University, Nanjing 210096, China; †††† College of Life Sciences, Northwest University, No. 229, North Taibai Road, Xian, Shaanxi Province, 710069, China

**Keywords:** LMICs, COVID-19 pandemic, mutations, infection waves, vaccination, genetic surveillance

## Abstract

**Background:**

Severe acute respiratory syndrome coronavirus-2(SARS-CoV-2) has infected over 100million individuals worldwide with diverse impacts on nations. The rising cases of new strains and resultant infection waves create an urgent need to assess the readiness of countries especially in Africa to mitigate the impact on community transmission. This paper delivers a brief synopsis of the novel SARS-CoV-2, emerging cases of new variants reported worldwide, and implications for genetic surveillance of disease transmission in low- and middle-income countries (LMICs) especially Africa.

**Materials and Methods:**

Literature search used keywords like SARS-CoV-2; COVID-19 epidemiology; pandemic waves; corona outbreak, clinical syndromes, treatments, prevention and control. Cross-sectional and observational studies published on COVID-19 from 2019 till date of study provided main information sources. Databases such as Web of Science, Embase, PubMed and Google Scholar were utilised.

**Main findings:**

Over 220 countries have documented COVID-19 cases with varied severity till date. Before the spikes in resurgence, a highly virulent mutated (>90% fatality rate) novel strain of COVID-19 had been documented. There is very little data to ascertain the impact of the COVID-19 infection waves in LMICs.

**Discussion:**

LMICs especially African countries still grapple with significant challenges like inefficient surveillance mechanisms, inadequate vaccination coverage, inadequate enforcement of environmental health strategies, poor health systems etc. Hence, Africa’s fate remains dicey in the face of the dynamic evolution of the SARS-CoV-2 and other identified challenges.

**Conclusion:**

The adoption of a multidisciplinary approach to mitigate the impact of emergence of mutant SARS-CoV-2 variants and resurgence of infection spike is recommended.

## Introduction

### Overview of the SARS-COV-2

From late December 2019 to early January 2020, several pneumonia cases of unknown origin were reported in Wuhan city of China and subsequently traced to a new coronavirus. The coronavirus disease-2019 (COVID-19) was named after the novel coronavirus. This scourge subsequently continued to infect people globally (Wuhan Municipal Health Commission, 2019). The degree of severity of the disease varies among individuals and regions (Ahmad *and* Rodriguez-Morales, 2019). The disease initially was spreading among animal populations especially bats and then moved to the human population (Ahmad *et al.*, 2020; Perlman, 2020). Despite considerable efforts to contain the disease in China, the virus has spread across the globe and thereby labelled a global public health emergency (Mahase, 2020).

However, due to its global expansion and infectiousness, the World Health Organization (WHO) raised the disease status to a pandemic of global concern in March 2020 (WHO, 2020a).

### COVID-19 and Africa

“The 54 countries of Africa recorded 100,000 cases in 90 days, but the number of cases became double in another 19 days while 300, 000 cases milestone was reached in another 12 days. As at 8^th^ July, 2020, the number of cases had skyrocketed to 500,000 and the death toll was 12,000. Although the number of cases in Africa rose sporadically to an average of 11,000 daily, these numbers portrayed the slower trend of the disease across the continent which came as a bit of relief (Wadvalla, 2020); given the already identified challenge of weak and over-burdened health system across African nations (Ohia *et al.*, 2020). Five countries (i.e. Algeria, Egypt, Ghana, Nigeria, and South Africa) accounted for seventy-one percent of COVID-19 cases recorded in Africa. And of these, South Africa contributed forty-three percent of the cases recorded on the continent.

In Sub-Saharan Africa, almost 91 percent of COVID-19 infections occurred in people under the age of 60, and over 80 percent of cases are asymptomatic (Adams *et al.*, 2021). Earlier at the onset of the pandemic, it was postulated that Africa would be hard hit by the COVID-19 challenge (Anadolu, 2020). However, recent trends of the disease have shown an opposite of the earlier postulations and this essentially raises questions that may require further scientific probing. Howbeit, it is important to note other reasons such as the fact that there is still paucity of data needed to understand why the numbers are not as high as originally predicted. The actual figures may however be more than currently recorded, since challenges in health care systems of low income nations make it difficult to assess exact numbers. In addition, Africa’s young population compared with the rest of the world could imply that continent probably has more asymptomatic carriers and transmitters of the disease. Thus accounting for the over 80 percent asymptomatic cases estimated earlier (Adams *et al.*, 2021).

### Transmission routes of SARS-CoV-2

Contact, aerosols, droplets, airborne, fomite, fecal-oral, blood-borne, mother-to-child, and animal-to-human transmission are all possible transmission routes for SARS-CoV-2. (Kumar *et al.*, 2020). The infection results into respiratory illness that could range from moderate to severe (fatal) disease manifestations and sometimes death. However, some infected individuals may remain asymptomatic i.e. never develop symptoms (WHO, 2020b).

SARS-CoV-2 can be transmitted directly or indirectly through contact with infected secretions like saliva, respiratory secretions or their respiratory aerosols that are possibly generated during coughing, sneezing, talking, or singing (Sheikh and Rabin, 2020). Transmission from respiratory aerosol occurs when an individual comes in close proximity (approximately 1 metre) to an infected individual already exhibiting respiratory symptoms such as sneezing and/or coughing; or an infected individual in such a close distance who could be singing or talking. Under such conditions, the risk of transmission becomes higher as the droplets from respiratory particles associated with the virus can get to the mouth, nose or eyes of any nearby person with weak immunity, thereby leading to infection. In addition, transmission through indirect routes involves the proximity of immunocompromised person to contaminated object/surface (fomite transmission) through touching of such surfaces thus enhancing the possibility of transmission of the infection.

Furthermore, due to the airborne nature of the disease, transmission can be facilitated when medical procedures resulting in release of aerosols are carried out i.e. “procedures that produces aerosol” (Bhaskar and Arun, 2020). The WHO has, in partnership with the scientific community, assessed and deliberated on the transmissibility of the COVID-19 infection through aerosols when there is no aerosol–generating activity, especially in indoor settings characterised by inadequate ventilations (Tang *et al.*, 2020). Additionally, the proximity of such settings possibly facilitates transmission from a small proportions of infected cases to a larger population (such as obtained in a super spreading situations), particularly in the absence of proper hand hygiene, usage of masks and observance of physical distancing (Dillon, 2020).

Respiratory secretions or aerosols released by infected persons could pollute surfaces and objects, resulting in fomites (polluted areas). Using Reverse Transcriptase–Polymerase Chain Reaction (RT-PCR) technique, it was found that active COVID-19 RNA could contaminate objects for a long period of time in relation to the environment, type of surface and ambient environmental conditions such as temperature and humidity (Zhou *et al.*, 2020a). These are especially so with particularly high concentrations in health institutions where SARS CoV-2 victims are being managed (Zhou *et al.*, 2020a). Hence, virus spreading can indirectly take place in a person via touching surfaces, materials or instruments polluted with this pathogen (e.g. stethoscope/thermometer), as the person touches the eyes, nose or mouth.

Transmission through fomite is rated as the most probable and efficient route in the of transmission of COVID-19. This is important, given findings relating infected cases’ close proximity to environmental contamination and the prior knowledge that other coronaviruses and respiratory viruses typically transmit in the same pattern.

Studies had found that the RNA of the SARS-CoV-2 was present in plasma or serum; also these pathogens reproduce in blood cells (Morris *et al.*, 2020). Moreover, the role played by blood-borne transmission cannot be ascertained; and small viral load in the blood shows that transmission risk via such medium can be negligible (Morris *et al.*, 2020).

The COVID-19 RNA is reportedly found in some biological samples, and also in faecal materials and urine of few sick persons (Guo *et al.*, 2021). Recent information reveals that people infected with COVID-19 could infect other mammals, in addition to dogs, cats, and domesticated mink (Oreshkova *et al.*, 2020). Nevertheless, it is not certain whether these infected mammals can result in risk of transmission to humans.

### Virology of the SARS-CoV-2

**Figure 1:**
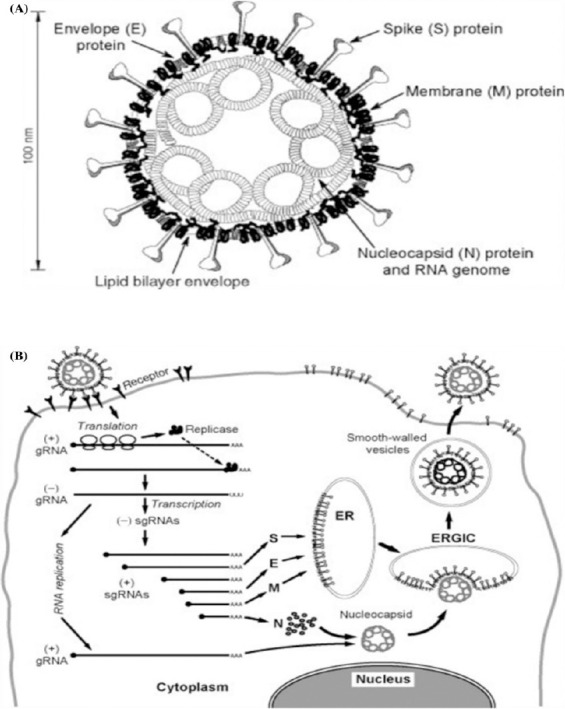
(A) Structure of coronavirus (B) Life-cycle (Masters, 2006).

COVID-19 is an extremely infectious and deadly condition whose etiology is SARS-CoV-2 virus, belonging to a group of viruses named coronaviruses. These Coronaviruses (CoV) were first reported in the mid-1960s and have been implicated as agents of disease in birds, mammals, and humans (Lee, 2015; Kumar *et al.*, 2020; Ohia and Uwalaka, 2021). They have led to widespread pandemics in the recent past decades (Morens *et al.*, 2020). These coronaviruses are from the family, *Coronaviridae* so named for the crown-like spikes found on their outer structure (Figures 1). Taxonomically, based on the classification by the International Committee on Taxonomy of Viruses (ICTV), coronaviruses belong to the order Nidovirales, Coronavirinae subfamily and *Coronaviridae* family.

Coronaviruses have a +ve sense, ss-RNA genome having a base length of 26 - 32 kilobases (kb) length. Therefore, having the largest genomes of RNA viruses (Abdoulaye, *et al.*, 2021); which are classified as alpha-CoVs, beta-CoVs, and gamma-CoVs (Carmine and Federico, 2020). The life-cycle of coronaviruses is presented in Figure 1b.

### The virus spread and its molecular mechanisms

Coronaviruses, which were illustrated by Tyrell and Bynoe (1966), are positive single stranded large RNA, enveloped viruses. They elicit infections in humans and many other animals; epidemiologically, males are more infected. This type of coronavirus is rated third, mostly causing severe scourge and pathology in man, though with less fatality than Severe acute respiratory syndrome (SARS) and Middle East respiratory syndrome (MERS). On 10 January 2020, the virus’ initial genome sequence was published on the Virological website, and two days later, on 12 January 2020, the GISAID database released other nearly full genome sequences reported by several research institutes (Virological.org, 2020; GSAID Database 2020). Out of the 7 coronavirus subtypes known to cause infection in man, the present COVID-19 belongs to the beta group of coronaviruses. It has 4 structural genes which code for membrane glycoprotein-M, spike protein-S, small membrane protein-SM, and nucleocapsid protein-N, among others. The virus was discovered to be identical (upto 96 per cent) to the genome of a bat coronavirus. Furthermore, recent researches involving 425 confirmed cases, has shown that the present epidemic may multiply two-folds in the population of affected individuals every seven days and that each infected individual can spread the infection to an average of 2.2 other persons (Velavan and Meyer 2020)**.**

**Figure 2:**
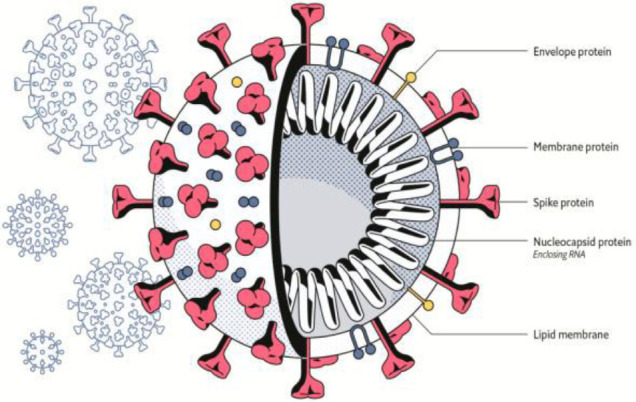
The structure of SARS-CoV-2 (CDC, 2020).

Coronaviruses are spherical and reasonably pleiomorphic (Figure 2). They measure 80–120 nm in diameter on the average. They possess a petal-shaped surface that extends between 17 and 20 nm from the exterior of the virion. The distal extremity of these coronaviruses has a miniature base that rises to a width of around 10 nm. Some may possess another cluster of projections, 5–10-nm long, beneath the major spikes as hemagglutinin-esterase (HE) protein (CDC, 2020). Other constituents are:

- S glycoprotein which is a very large anchor attachment to the receptor including viral binding and host cell boundary by inserting into the endoplasmic reticulum.
- M glycoprotein (Membrane protein) is the richest constituent and provides virion envelope with its shape.
- The Envelope protein (E protein) is a miniature polypeptide, measuring 8.4 to 12 kDa (76–109 amino acids), this is a negligible component of virion.
- The N protein or Nucleocapsid protein, measuring between 43 to 50 kDa, is the phosphoprotein part of the helical nucleocapsid that is believed to join the genomic RNA in a beads-on-a-thread manner.

The SARS-CoV-2 S-protein is about 1,273 amino acids long. There is seventy-three percent similarity between the amino acids of the SARS-CoV and SARS-CoV-2 contained in the receptor-binding domain (RBD) of the S-protein. The occurrence of 4 amino acid residues (PRRA) at the intersection of the S1 and S2 subunits of the S protein is another genetic feature unique to SARS-CoV-2 (Liu *et al.*, 2020). This insertion provides a polybasic cleavage site (RRAR) that increases the effectiveness of furin and other proteases. This type of S1–S2 cleavage region is non-characteristic of other associated viruses from the subgenus Sarbecovirus, with the exception of a comparable RmYN02, a bat-derived coronavirus, with three amino acid insertions (PAA), that was recently documented from *Rhinolophus malayanus* in China (Zhou *et al.*, 2020b). Despite the fact that the insertion in RmYN02 does not actively represent a polybasic cleavage region but supports the opinion that this characteristic and unique feature of the COVID-19 is contracted naturally (Zhou *et al.*, 2021).

Furthermore, previous report posited that structurally, the furin-cleavage region may decrease the stability of COVID-19 protein S necessary to join the receptor-binding domain (RBD) with its receptor (Arbeitman *et al.*, 2020). It still remains to be ascertained if increased COVID-19 transmissibility in comparison to other SARS-CoVs is a functional gain related to its possession of furin-like cleavage region.

The genomes of coronaviruses are non-segmented, single-stranded mRNA molecules. This RNA is infectious when transfected into host cells (Masters, 2006). Following that, a series of events occur that result in the nucleocapsid being deposited in the cytoplasm. The viral genomes become available for translation at this point. Membrane-bound structural proteins (M, S, and E) are delivered into the Golgi intermediate compartment of the endoplasmic reticulum.

Nucleocapsids are generated from the encapsidation of progeny genomes by the N protein, which merge with membrane-bound components for virions creation (Masters, 2006). These virions are then transported out of infected cells in smooth-walled vesicles, or Golgi sacs, to the plasma membrane. At the surface of the cell, S protein may also prompt the fusion of an infected cell with a nearby uninfected cell, causing the development of huge, multinucleate syncytia (Masters, 2006). When this happens, the virus spreads independent of the extracellular virus and this situation will likely escape from immune surveillance.

COVID-19 is phylogenetically related to bat viruses that cause SARS, showing that bats are the most likely primary reservoir (Kumar *et al.*, 2020). Despite the fact that the source of origin and transmission to humans is unknown, the rapid human-to-human transmission has been demonstrated. The 7 sub-groups of the human coronaviruses are: HCoV-229E (α-coronavirus), HCoV-NL63 (α-coronavirus), HCoV-OC43 (β-coronavirus), HCoV-HKU1 (β-coronavirus), MERS-CoV (β-coronavirus), SARS-CoV (β-coronavirus), and the newly discovered SARS-CoV2 (CDC, 2020; Ohia *et al.*, 2020).

### Uniqueness of the SARS COV-2 and its impact on the transmission of the COVID-19 infection

The SARS-CoV-2 possesses greater basic reproduction number (R_0_) than SARS-CoV-1, which makes transmission and pathogenicity of the COVID-19 disease unique as this characteristic multiplication rate explains its more efficient spread (Harrison *et al.*, 2020). Several other uniqueness of COVID-19 can help elucidate the improved transmission of the infection. Despite the fact that the two viruses prefer to act together with the angiotensin-converting enzyme 2 (ACE-2) receptor, SARS-CoV-2 has variations in the structure of its superficial proteins, which allows for tougher fastening to the ACE-2 receptor (Arbeitman *et al.*, 2020) and higher efficiency at avoiding host cells (Harrison *et al.*, 2020). SARS-CoV-2 also has a higher affinity for the upper respiratory tract and conjunctiva (Wölfel *et al.*, 2020), allowing it to easily infect the upper respiratory tract and coordinate airways (Varghese *et al.*, 2020).

The pandemic’s impact is centered on the transmission pathway and dynamics of circulation of the disease. Unlike other coronaviruses, the initial transmission pathway of SARS-CoV-2 is through infected aerosols from respiration. Infection from virus takes place through direct or indirect closeness to respiratory particles from nasal, conjunctival, or oral mucosa, during inhalation or lodgments on the mucous membranes (Varghese *et al.*, 2020). Target receptors from host, located primarily within the oropharynx and upper airway, are incorporated within the respiratory tract epithelium of humans. In addition, the ocular and gastrointestinal systems are vulnerable to infection and possibly operate as pathways for infection transmission (Varghese *et al.*, 2020).

Variables such as pattern of contact, ambient surrounding, host infectivity and socio-economic factors influence transmission risk (Harrison *et al.*, 2020). Majority of transmission takes place via close regional contact (e.g. 15 minutes direct contact within a range of 2 m) between individuals (Kumar *et al.*, 2020). Virus transmission is mainly proficient within households and via family and friends’ gatherings (Harrison *et al.*, 2020). Follow-up infection rates of household (i.e. the percentage of persons who contract the infection among cluster of vulnerable persons found to be with an initial case) fall within 4% to 35% (Harrison *et al.*, 2020). While living and sleeping under the same roof as, or partners of an infected individual promotes infection risk, lower infection risk is achieved by the quarantine of persons infected so as to limit contact with family (Favas *et al.*, 2020).

Additionally, other risk factors comprise eating in proximity with persons already infected, food sharing, and participating in activities involving large number of people (Favas *et al.*, 2020). Also, infection risk is significantly higher in enclosed environments as against outdoor settings (Harrison *et al.*, 2020). Furthermore, transmission by aerosol could still feature during lengthened presence in populated areas, less aerated enclosed settings (i.e. infection may take place at a range of >2 m) (CDC, 2020).

### Mutations and variants

Viruses, by virtue of their structure, are known to mutate very fast. The SARS-CoV-2 proved this phenomenon more than any other virus in history. To date, it has been accumulating mutations at a pace of one to two per month which implies that many of the genomes sequenced most recently vary at about 20 points from the earliest genomes sequenced in China in January, 2020 (Flores-Alanis *et al.*, 2021). A variant with a D614G mutation within the gene encoding the spike protein was discovered in 2020. Within months, the D614G mutation had substituted the first strain discovered in China, and by mid-2020, this strain had become the predominant version of the virus revolving around the world. A novel variant strain of SARS-CoV-2 containing a series of mutations has been recently described in the United Kingdom with high prevalence in London and South-East England (Flores-Alanis *et al.*, 2021).

The surge in cases recorded in Kent, South- East England in early December, 2020 was attributed to the strain named B.1.1.7. (Volz *et al.*, 2021). This variant has acquired 23 mutations all at once of which eight were on the viral surface, within the gene encoding the spike protein, a feat the scientists never seen before. Within a short period, less than a fortnight, that particular variant has led to a lot of havoc in the United Kingdom and several other places in Europe. It is postulated that this viral mutation may have occurred in an immunocompromised individual(s) (Volz *et al.*, 2021). The novel coronavirus is capable of correcting errors when it replicates and hence tends to possess a fairly stable genome distinct from the common flu virus. The weakened immune systems may be due to people placed on immunosuppressant drugs or undergoing chemotherapy. The usage of the word “variant” is appropriate, as viruses subsist as a collection of variants referred to as “quasi-species” and at the time of infection, particular viruses are picked. As multiplication occurs, the virus regenerates broad variations but retains its similarity to the originally infecting virus. This similarity has been acknowledged and thus the apprehension that a “new strain” is emerging.

It is important to note that a novel strain cannot dominate the established virus except it offers certain qualities and benefits over the virus. These features have not been documented so far. Another reason may be that an unintended variant will most probably arise in the regions of the globe where infection is widespread, not the United Kingdom (Science Media centre 2020). These variants appear to be spreading between people. Two of these - N501Y and 69-70del were found worrisome since these attach to the angiotensin-converting enzyme 2 receptor, which is the point of entry into human cells. The two amino-acid deletion 69-70 Delta, was first detected independently in a patient undergoing treatment with immune-suppressants i.e. remdesevir, convalescent plasma and neutralizing antibodies. However, the patient passed on after few months (Science Media centre 2020; Andreano *et al.*, 2020). Although the virus did not possess this deletion previously, it developed it over time. This variant can easily be detected by PCR tests called TaqPath which is used widely in the United Kingdom (UK). This test is faster and also cheaper. Such mutants may evade antibodies meant for conventional viruses which may be a problem for control.

Some mutant variants different from the UK variant were also reported from South African Provinces where cases were rising, Spain, and the Netherlands. The symptoms of both the original and the variants remained the same (e.g. continuous cough, chest pains, fever, loss of taste and smell, headache, tiredness, muscle pain, diarrhea, confusion, skin rash). Further data are needed before any conclusions are drawn (Kupferschmidt, 2020). More challenging issues may arise as we witness increase in natural immunity (herd immunity) level as a result of increasing deployment of vaccines, drug therapies, and non-pharmaceutical interventions. These have the capacity to cause a variety of genetic modifications in the virus and to elicit other harmful variations of mutations of the virus.

Furthermore, in India, a Corona variant was detected which is spreading very fast. It is named N440K. This strain is suspected not to be affected by the SARS-CoV-2 antibodies. About 272 samples collected from a southern state Andhra Pradesh, when examined, showed contamination with the new variant at 34% (Personal communication, December 28, 2020). Also, SARS-CoV-2 mink-associated variant strain has been reported in Denmark. Up till now, six nations (Denmark, Netherlands, Spain, Sweden, Italy and United States of America) have documented SARS-CoV-2 in domesticated minks to the World Organisation for Animal Health (OIE). However, none has yet been recorded from Africa (Oreshkova *et al.*, 2020; Flores-Alanis *et al.*, 2021).

### Virus mutations: Genetic diversity, SARS-CoV-2 transmission, pathogenicity and implications

Generally, RNA viruses with the inclusion of the SAR-CoV-2 possess increased mutation rates. These are prominently associated with the heightened virulence of the viruses and their ability to evolve (Meyerowitz *et al.*, 2020). Mutation in the S-protein of the virus poses a huge clinical and public health concern because of its potential to modify the tropism of a virus, together with adaptation of the virus to novel hosts and intensification of its pathogenesis (Varghese *et al.*, 2020). Hence, identifying and understanding mutations within the S protein from various nations and territories will help the provision of explanations for the consistent shift in its structure. This will possibly shed more light on the mechanisms through which these mutations facilitate varying transmissions of SARS-CoV-2 in various areas of the globe. Despite this, there is still little knowledge of the extent to which the S protein mutation-mediated spread of SARS-CoV-2 is dependent on variables such as race, ethnicity, or geographic location of individuals.

For example, it has been documented that the genomic sequences of the novel isolates that originated from China are closely linked to the ones circulating within the United States and regions of Europe (Reference). In spite of these, the SARS-CoV-2 virus has gone through numerous mutations that have led to the suggestions that the ongoing mutations could result into more serious and virulent COVID-19. It is possible that the strains being circulated in the sub-Saharan Africa area could have originated from the earlier circulated virus present in the beginning of the COVID-19 pandemic that may have gone through minimal or no mutations. As an example, the earliest SARS-CoV-2 sequenced from Africa showed a phylogenetic relationship to early isolates obtained from Wuhan (Yang *et al.*, 2020).

The first circulated strains were the S-types which were less virulent (Borkakoty and Bali, 2021). Therefore, stricter quarantine measures are needed for individuals with recent international travel history, within a fortnight, especially to regions or areas with lower incidence and case fatality rates.

### The role of community transmission in mutation of the virus

As at the 8^th^ and 9^th^ months of the year 2020, a variant of SARS-CoV-2 related to infections recorded in farmed minks which consequently spread to humans, was recognized in Denmark. This variant, known as the “Cluster 5” variant by Danish investigators, possesses a mutation blend not formerly documented. Subsequently, towards the middle of the year 2020, a variant named SARS-CoV-2 VOC 202012/01 (Variant of Concern year 2020, month 12/ variant 01) was described to WHO by the UK. The variant is made up of substitution of twenty-three nucleotides and not related phylogenetically to the virus previously transmitting in the United Kingdom before the variant was found.

Where and how COVID-19 VOC 202012/01 came from remains a mirage. COVID-19 VOC 202012/01 earlier came up in south-eastern region of England and within a short period of time started replacing some related viruses around this region including London (Flores-Alanis *et al.*, 2021). By the end of year 2020, COVID-19 VOC 202012/01 was recognized from genomic testing and routine samplings carried out all over the United Kingdom (Flores-Alanis *et al.*, 2021). The United Kingdom government of the affected nations were carrying out virological and epidemiological surveillance to advance measurement of transmission capability, risk of re-infection, ruthlessness including response to antibody towards the novel variants. One of the mutants (N501Y) was located in the two variants in the fastening domain receptor.

SARS-CoV-2 mutates frequently, obtaining about one new mutation in its genome fortnightly (CDC, 2020). Many of these mutations are silent (causing no variations in the structure of the encoded proteins) due to the fact that they produce a three-letter codon that turns to the same amino acid (this implies that they are “synonymous”). Other mutations may transform the codon in a manner that results to an amino acid change (meaning that they are “non-synonymous”), however this amino acid substitution will not influence the protein’s function (CDC, 2020).

Furthermore, new cases of the SARS-CoV-2 variations have been connected to farm-raised minks with 12 instances of a single version reported in November, 2020 in North Jutland, Denmark (Fenollar *et al.*, 2021). The age range of reported cases was seven to seventy-nine years; eight of the cases had a connection to the mink farm industry and four of the cases emanated from the immediate areas of the farms. Preliminary investigations proposed that the clinical signs, severity and spread pattern among those infected are comparable to other revolving SARS-CoV-2 viruses (Fenollar *et al.*, 2021). On the other hand, this current variant (known as the “cluster 5” variant), is reported to have a combination of mutations, or alterations which were not previously described. Again, this implies that the identified changes in this variant are yet to be well documented.

The implications of the emergence of these new variants and consequences of these mutations will include the following based on the report by CDC, 2020:

- *High efficiency of spread in human population*. There is at present data that one mutant, D614G, possesses the ability and feature to quickly spread. And under laboratory conditions the G614 variants propagate very efficiently within human respiratory epithelial cells, thus out-competing the D614 viruses. In addition, it has been documented that the G614 variant transmits faster than viruses lacking the mutation.
- *Capability of causing mild to very severe disease manifestation* *in humans*. There is no evidence yet to suggest that VOC 202012/01 can elicit very severe illness than other SARS-CoV-2 variants.
- *Capacity to elude discovery* *by target-specific diagnostic tests*. Commercially available polymerase chain reaction (PCR) tests mostly possess multiple targets such that their ability to detect the virus is enhanced and in the eventuality that a mutation affects one of the targets, the remaining PCR targets can still function.
- *Reduced vulnerability to beneficial agents* like *monoclonal antibodies*.
- *Ability to avoid vaccine-induced immunity*. Vaccines authorized by the FDA are “polyclonal,” thus produce antibodies that target many portions of the spike protein. This then implies that the virus may probably require an accumulation of multiple mutations in the spike protein to enable its effective avoidance of vaccine-induced immunity or that developed by the cycle of natural infection.

Amid the scenarios above, the ability to avoid vaccine-induced immunity will most probably be the most disturbing.

This is because as more people are vaccinated, immunological pressure builds up, which may unwittingly push, amplify, and speed up the appearance of such variants through selection for “escape mutants.” However, there is currently no proof that this is happening, and most experts believe that given the virus’ nature, these “escape mutants” are unlikely to emerge.

### Understanding the spikes and infection/transmission waves

Many countries have reported spikes in infection cases and also in COVID-19 pandemic- associated mortalities. What does a spike indicate? Some people misinterpret the concept by postulating that as more testing is carried out infection numbers spike. But it is totally wrong and confusing. It is obvious that when people do not follow the stipulated COVID-19 control guidelines, e.g. wearing of facemasks, hand washing, isolation and social distancing, travel, etc., the infections rise disproportionately. Seasonal variations are also ruled out, as there have been no seasonal-related trends observed in several countries where infections were high. However, in the weeks up to early December, 2020, the increase in rates were mainly in North Africa, specifically Morocco, where temperatures have been falling as winter approached. The Africa CDC had earlier reported the start of the second wave of the pandemic in Africa. There have been three main trajectories in African countries:

(i) Countries where the infection transmission curve never flattened, or where recorded case numbers were low until August when they peaked significantly;
(ii) Countries where the infection transmission curve flattened after cases peaked in July, but are now seeing another rise in numbers; and
(iii) Countries that have had a consistent decline in cases over time (albeit after an initial rise in cases).

Many countries quickly reopened public spaces after the initial closure. However, in many locations, the proportion of the population infected with the coronavirus remained high, and virus transmission was rekindled when individuals intensified their activities and intimate contact with one another. Similar observations have been made while reopening public institutions such as schools, educational institutions and work places across the world. It is therefore important to understand the infection wave phenomenon in the context of the ongoing pandemic.

### What is an infection wave? Experiences from countries across the globe

This is an occurrence of infections which can come up in a pandemic. A set of people will be infected first after which the infection level comes down but later on, the infection increases in different parts of the population thereby leading to second wave of infection (Cooper *et al.*, 2020). Over 220 countries have documented COVID-19 cases till date. Of these, one hundred and twenty countries have documented clear second waves or late first waves starting from late 2020. Of these countries that have distinctly experienced the second wave or late first wave, six clearly came out of their second infection waves (i.e. Australia, South Korea, Japan, Hong Kong, Vietnam and Singapore). Vietnam and Hong Kong had equivalent achievements in containing the second wave and in the subsequent summer the number of cases dropped drastically (The conversation, 2020). Israel’s experience was different as the second wave started much earlier and was more severe than the first. This was majorly due to transmission among in-school individuals (high school and middle-school students) and mishandling of the first lockdown. In addition, the enforcement of face mask use in the country was limited during the summer alongside the moderate restrictions put in place. The country took a while to move from the second wave with a range of 50-90 new cases per day.

### Implications of the second wave and mutating strains of the virus on Africa, her health systems generally

Approximately 2.7 million Africans were infected with COVID-19, with mortality over 65,000 documented on the continent in the year 2020. An increase in incidence in the fourth quarter of the year, in addition to the emergence of more contagious mutations, accumulates to present newer challenges for Africa going forward. On the average, 28 countries in Africa have already documented rising weekly cases of new COVID-19 infections in comparison to the previous weeks since the beginning of October, 2020. These progressions have led to an estimated average of 22,000 new cases per day in December, 2020, thus overriding the peak rate of 18,000 recorded in the first wave in July, 2020. Approximately, about half of the total number of cases recorded on the continent had been documented since October 2020 as more cases continue to be reported in South Africa and Morocco, countries with the best capacity for testing in Africa. However, the levels of reported cases are mostly representative of what has been observed within Africa. Countries like Tunisia, Botswana, Uganda, Angola, Eritrea, and Burkina Faso, have experienced sharp rises in transmission during the second wave, although their cumulative number of reported cases remains quite minimal. The modifications of the virus strains isolated in the UK and transported to South Africa in December 2020 have a substantial transmissibility potential. As a result, there’s a better chance that the second wave of the infection may spread further. For instance, the mutated strain has been implicated as dominating and facilitating the second wave in South Africa. In addition, it is pertinent to keep in mind that the second and or subsequent waves of the most recent pandemic - the Spanish flu pandemic (which occurred over a century ago) was widely distributed and deadly in Africa (and other parts of the globe) than its first wave. The implication of this is that the virulence of these new COVID-19 variants is still quite unpredictable. It is hoped that this second and subsequent SARS-CoV-2 infection waves will not behave in the same manner as depicted for Spanish flu pandemic in Africa.

### Africa’s challenges in the face of the COVID-19 pandemic

At the beginning of the pandemic, governments of countries within sub-Saharan Africa took proactive steps to enact strategies such as restrictions of travels and total lockdown. Most countries activated their incident management response mechanisms, and mobilized front-line healthcare personnel for training. However, within a few months, it was realized that human resources in sub-Saharan Africa was grossly unprepared for the health challenge due to several reasons including: lack of capacity for testing, isolating confirmed or assumed cases, trace contacts, and manage infected individuals having severe illness, inadequate diagnostic kits, poor healthcare systems, grossly inadequate intensive care units, ventilators, shortage of health professionals (Ohia *et al.*, 2020). This is in addition to other bottlenecks such as incessant industrial actions and health workers’ strikes, increasing SARS-CoV-2 infections among healthcare and frontline caregivers and professionals, limited access to personal protective equipment (PPEs), health maladministration and corruption. In addition, environmental response data is lacking and practically non-existent in most African countries. Africa is currently bedevilled with conflicts and unrests amidst the pandemic leading to riskier behaviours and non-adherence to safety precautions in the control of the disease (Ohia and Salawu, 2020). People affected by COVID-19 and living in precarious environments, therefore, need special attention. Such situations may be in overcrowded conditions, on the streets, in makeshift camps or substandard housing. Under these situations, most conventional or recommended procedures are a luxury and not practicable. It is vital to note that these groups of the population are already in poor health and excluded from the formal healthcare system. For instance, where one finds a seriously ill COVID-19 patient, such a patient will need oxygen along with the proffered treatments. On the average, about 80 per cent of people hospitalised for COVID-19 need between 3 and 15 litres of oxygen per minute while for the remaining 20 per cent, the needs are more severe as they usually will need more than 20 litres per minute. These needs are usually very difficult to meet in the context of the current challenges facing Africa’s health systems.

### Way forward

**Figure 3:**
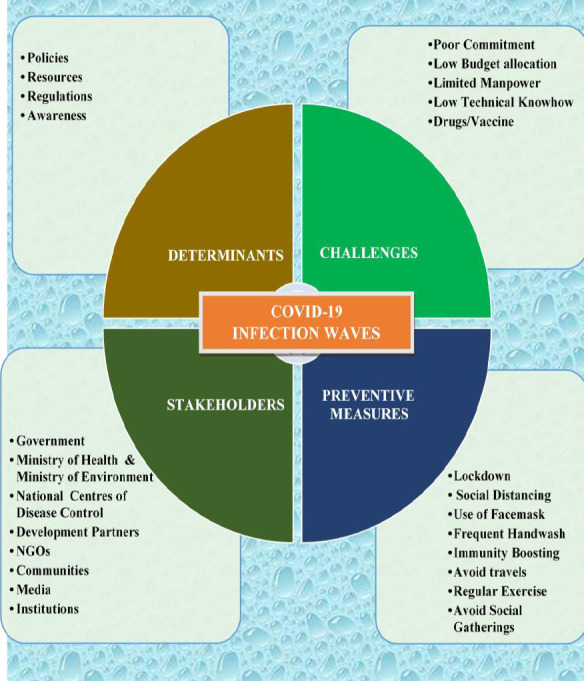
Conceptual diagram of COVID-19 infection wave multi-dimensional management approach

The emergence and re-emergence of highly infectious diseases are posing significant public health risks that pose significant threat to human populations as well as animals all around the world (Munaj *et al.*, 2017; Dhama *et al.*, 2020). In addressing the COVID-19 pandemic, there is the need for a multi-dimensional approach in tackling the impact of the highly virulent variant(s), the ongoing spikes, and resultant infection wave occurrences. This approach will require the understanding of the inter-relatedness of all factors that can control the second wave and also consolidate the efforts at curtailing the impact of the disease and the second wave already reported across several countries in Africa (Figure 3).

The WHO published a provisional guideline on 16 April 2020 that advises on Public Health and Social Measures (PHSM) adjustment to manage the risk of resurgence. Several annexes were developed to support various measures in various contexts by helping countries, policy makers and stakeholders involved in the development and operationalisation of procedures to control the possibilities of workplace-associated COVID-19 transmission. Other types of labor unions, business organizations, public and employment authorities, and health workers are also included.

Furthermore, this document provides guidance for workplaces and employees in non-health environments. For specialized workplaces, additional safeguards may be necessary. In addition, recommendations exist in the current WHO Guidelines to ensure the safeguard of the health and safety of individual front-line health workers in public facilities such as detention centers, schools, food ventures, aviation industry, water, sanitation, waste management, camps and construction (WHO, 2020c; Bender, 2020; WHO, 2020d).

In line with the foregoing, it will be very beneficial for countries to activate and strengthen their environmental health laws, enforcement of safety, health and environment policies in workplaces, public places such as religious and recreational centres in addition to collaborative engagements by all professional stakeholders in the fight against infectious diseases such as COVID-19.

### Recommendations

In order for African countries to understand and prepare for the emergence of these variants and to guide against community transmission of SARS CoV-2’s eventual mutants, the national authorities must intensify efforts to carry out epidemiological surveillance. This is in order to assess the behavior and extent of spread of the new variants in countries outside Africa. National research teams must study the mutation effects on re-infection potential, diagnostic testing, severity of infection, provision of vaccine, and transmission capability. Government including researchers should work together with WHO COVID-19 evolution working group to evaluate epidemiological, phylogenetic, laboratory investigations and modeling reports as soon as their findings are ready.

Evaluation and investigation of the genetic variety and mutations in SARS-CoV-2 will be highly beneficial to environmental health researchers, epidemiologists and clinicians working assiduously in Africa to monitor the characteristic patterns of this pandemic as it is observed in developed world. Although the reported mortality and morbidity of SARS-CoV-2 from Africa is not comparable with what is obtainable in western countries presently, African scientists need to build capacity to enhance their understanding of the pathways of the genetic diversity and mutations. Also important is how this impacts the distribution and the pathogenesis of SARS-CoV-2. In order to avert importation of new strains from affected countries outside Africa by putting control strategies in place.

Activities such as community engagement and risk communication must be upgraded to elucidate the consequences of SARS-CoV-2 variants to the community wellbeing and to lay emphasis on the significance of ensuring current precautionary measures to control the virus spread. These should include wearing face masks, practicable hand hygiene, keeping physical distance, cough manners, respiratory etiquette in general, provision of good ventilation and keeping away from populated or jam-packed environments. African countries must therefore put in proactive efforts to contain the SARS-CoV-2 strains circulating in some countries outside the continent.

## Conclusion

SARS-CoV-2 mutations have very distinct relevance in COVID-19 pathogenicity, the improvement and development of diagnostics tools (especially sero-diagnostics), antivirals, and vaccines. Hence, for SARS-CoV-2, multidisciplinary efforts must be strengthened in order to facilitate the rapid documentation of new mutants in Africa, their impact on available interventions and COVID-19 control measures ahead of time.

### Ethical Approval:

Not applicable.

### Conflicting interests

All authors declare that they have no known conflicting personal or financial interests which may have impacted the development of this work.

**List of Abbreviations:**
ACE-2: Angiotensin-converting enzyme-2
CDC: Centre for Disease Control
COVID-19: Coronavirus disease 2019
DNA: Deoxyribonucleic Acid
FDA: Food and Drugs Administration
HE: Hemagglutinin-esterase
ICTV: International Committee on Taxonomy of Viruses
LMICs: Low- and middle-income countries
MERS: Middle East respiratory syndrome
OIE: World Organisation for Animal Health
PCR: Polymerase Chain Reaction
PHSM: Public Health and Social Measures, R_0_Reproduction number
RBD: Receptor-binding domain
ssRNA: Single stranded Ribonucleic Acid
SARS: Severe acute respiratory syndrome
SARS-CoV-2: Severe acute respiratory syndrome coronavirus-
SM: Small membrane protein
UK: United Kingdom
WHO: World Health Organization
